# Assessment of Agroeconomic Indicators of *Sesamum indicum* L. as Influenced by Application of Boron at Different Levels and Plant Growth Stages

**DOI:** 10.3390/molecules26216699

**Published:** 2021-11-05

**Authors:** Salwinder Singh Dhaliwal, Vivek Sharma, Arvind Kumar Shukla, Vibha Verma, Sanjib Kumar Behera, Prabhjodh Singh Sandhu, Kamaljit Kaur, Ahmed Gaber, Yusuf S. Althobaiti, Abdelhadi A. Abdelhadi, Akbar Hossain

**Affiliations:** 1 Department of Soil Science, Punjab Agricultural University, Ludhiana 141004, Punjab, India; ssdhaliwal@pau.edu (S.S.D.); sharmavivek@pau.edu (V.S.); vermavibha@pau.edu (V.V.); 2 Indian Institute of Soil Science, Bhopal 462038, Madhya Pradesh, India; arvindshukla2k3@yahoo.co.in (A.K.S.); sanjibkumarbehera123@gmail.com (S.K.B.); 3 Department of Plant Breeding and Genetics, Punjab Agricultural University, Ludhiana 141004, Punjab, India; prabhsandhu@pau.edu; 4 Department of Food Science and Technology, Punjab Agricultural University, Ludhiana 141004, Punjab, India; kamalbhella@pau.edu; 5 Department of Biology, College of Science, Taif University, P.O. Box 11099, Taif 21944, Saudi Arabia; 6 Department of Pharmacology and Toxicology, College of Pharmacy, Taif University, P.O. Box 11099, Taif 21944, Saudi Arabia; ys.althobaiti@tu.edu.sa; 7 Genetics of Department, Faculty of Agriculture, Cairo University, Giza 12613, Egypt; abdelhadi.abdallah@agr.cu.edu.eg; 8 Department of Agronomy, Bangladesh Wheat and Maize Research Institute, Dinajpur 5200, Bangladesh

**Keywords:** sesame, seed yield, B uptake, theoretical oil content, antioxidant activity, acid value, agronomic efficiency indices

## Abstract

To achieve the nutritional target of human food, boron (B) has been described as an essential mineral in determining seed and theoretical oil yield of *Sesamum indicum* L. The research to increase its cultivation is garnering attention due to its high oil content, quality and its utilization for various purposes, which include human nutrition as well as its use in the food industry. For this, a two-year field experiment was performed at PAU, Punjab, India to determine the effect of different concentrations of foliar-applied B (20, 30 and 40 mg L^−1^) and different growth stages of crop, i.e., we measured the effects on agroeconomic indicators and certain quality parameters of sesame using different concentrations of B applied at the flowering and capsule formation stages as compared to using water spray and untreated plants. Water spray did not significantly affect the studied parameters. However, B application significantly increased the yield, uptake, antioxidant activity (AOA) and theoretical oil content (TOC) compared to those of untreated plants. The maximum increase in seed yield (26.75%), B seed and stover uptake (64.08% and 69.25%, respectively) as well as highest AOA (69.41%) and benefit to cost ratio (B:C ratio 2.63) was recorded when B was applied at 30 mg L^−1^ at the flowering and capsule formation stages. However, the maximum sesame yield and B uptake were recorded when B was applied at a rate of 30 mg L^−1^. A significant increase in TOC was also recorded with a B application rate of 30 mg L^−1^. For efficiency indices, the higher values of boron agronomic efficiency (BAE) and boron crop recovery efficiency (BCRE) were recorded when B was applied at 20 mg L^−1^ (5.25 and 30.56, respectively) and 30 mg L^−1^ (4.96 and 26.11, respectively) at the flowering and capsule formation stages. In conclusion, application of B @ 30 mg L^−1^ at the flowering and capsule formation stages seemed a viable technique to enhance yield, B uptake and economic returns of sesame.

## 1. Introduction

Sesame (*Sesamum indicum* L.), a popular health food, has been growing in Asia and Africa for over 7,500 years. Sesame seed contains oil (46–64%), protein (15–25%), carbohydrates (20–25%) and micronutrients. Nutritionally, sesame seeds are rich in oil with high levels of unsaturated fatty acids, mainly oleic and linoleic, protein, especially high levels of methionine, and micronutrients such as minerals, lignans, tocopherol and phytosterol; thus, sesame is known as the ‘queen of oilseeds’ [1]. However, due to the pronounced antioxidant activity of its seed oil, sesame offers a high shelf life and is also known as the ‘seed of immortality’ [1]. Owing to the high nutritional and therapeutic values, sesame is also used extensively in confectionaries and baked goods globally [2]. In India, sesame is grown over an area of 1.85 million ha and 0.83 million tonnes of sesame grains are produced yearly with an average productivity of 474 kg ha^−1^ [3]. Since sesame yield is influenced by various physiological processes occurring in the plants, it can be further changed by management practices in a given environment [4]. 

Inadequate management practices, along with intensive use of fertilizers, have resulted in an imbalance of micronutrients in the fertile regions of Northwest India [5]. The insufficient availability of a particular essential micronutrient may alter plant growth and thus reduce crop yield [6]. The essential role of micronutrients in crop productivity has been reported in the literature [7]. Micronutrients are needed in small quantities by plants; these elements play a crucial role in plant development [8]. Boron (B) is known as an essential micronutrient required for plant growth and its deficiency is the most globally widespread limit to crop production both quantitatively and qualitatively. It is well known for its role in pollen production and the viability of pollen tubes as well as their germination and growth. The limited supply of B is a major constraint in oil production and impairs its quality [9]. It is also involved in the synthesis of lignin, which provides strength to the cell walls of biological membranes [10].

Micronutrient enrichment of crops through agronomic biofortification (in soil or foliar) has been evidenced to ameliorate the adverse effects of micronutrient deficiencies and improve the yield and nutritional parameters of the crops [11]. Foliar fertilization has gathered much attention in recent years due to the high water solubility of fertilizers. Foliar application is more effective when the absorption of nutrients in the roots is insufficient due to low soil moisture, and foliar fertilization is also a better option when the crop height is greater [12]. Exogenous supply of nutrients through foliar facilitates a quick regain in a drought situation and also prevents nutrient losses in soil [13]. 

The improved oil quality at a B concentration of 30 mg L^−1^ has been reported. On the other hand, when using foliar B application at 20 mg L^−1^ the maximum results for growth parameters including plant height, plant stem diameter and capsule length were recorded as compared to those of the control or those with treatment using a higher B concentration [14]. In another report, the highest values of seed and stover yield were recorded using a treatment of 0.15% foliar spray of B in sesame due to fertility improvement and the translocation of photosynthates [15]. Studies in the literature have demonstrated that the seed yield of sesamum can be increased with an increase in B levels [16,17]. For instance, a significant increase of 10.7% in seed yield of sesame has been recorded with 0.15% B application over the control under red laterite soils [18]. There is also evidence that boron application can improve certain quality parameters of oilseed crops. Furthermore, the oil content of some sunflower cultivars has been found to increase with B application [19]. In another study, the activity of several antioxidant enzymes in maize [20] and soybeans [21] have improved with B application. Thus, the present study was conducted to evaluate the effect of B on sesame yield, B uptake, certain oil quality parameters and the economics of sesame cultivation.

## 2. Materials and Methods

### 2.1. Site Specification

The experiment was initiated in the kharif seasons of 2019 and 2020 (June to September) in sandy loam soil (Typic Ustochrept) at the Research Farm Area, Department of Soil Science, PAU, Ludhiana, India. The initial soil properties of the experimental field have been given in Table 1. 

### 2.2. Brief Description of the Methodology for Analysis of Soil Samples

Among soil characteristics, pH, EC and bulk density were estimated using the method reported by Jackson [22]. The pH and EC of the soil samples were estimated using a pH meter and an EC meter, respectively, whereas bulk density was estimated by employing the weighing bottle method. The OC content in soil was estimated using the wet combustion method [28]. The available N, P and K were determined using the alkaline KMnO_4_ method, the Olsen extractable P method and the neutral ammonium acetate method, respectively [22,23,24,25]. Diethylene triamine penta acetic acid (DTPA) extractable soil micronutrients viz. Zn, Cu, Fe and Mn were determined using DTPA-TEA buffer in the ratio of 1:2, and then their concentration was estimated using an atomic absorption spectrophotometer (AAS) [26]. The total P, total K and micronutrients in the soil were determined by digesting the soil samples with diacid, i.e., HNO_3_ and HClO_4_ in the ratio of 9:4, and these digests were analyzed for total extractable P, K and DTPA soil micronutrients after the appropriate dilutions. The available Zn, Cu, Fe and Mn contents were measured using AAS (Varian AAS FS 240 Model). 

### 2.3. Treatment Details and Estimation of Yield 

The experiment comprised of nine treatments viz., T_1_, control (no spray); T_2_, water spray at the flowering stage; T_3_–T_5_, foliar spray of B at 20, 30 and 40 mg L^−1^ at the flowering stage; T_2_ and T_6_, water spray at the flowering stage and capsule formation stages; T_7_–T_9_, foliar spray of B at 20, 30 and 40 mg L^−1^ at the flowering and capsule formation stages (Table 2). 

Borax (Na_2_B_4_O_7_·10H_2_O) containing 11.3% was used as a source of B for foliar application. The recommended dose of nitrogen was applied as basal at the time of sowing through urea. The sesame variety Punjab Til No. 2 was used for the experiment under the irrigated system with a plot size of 6 m by 4 m. The sowing was performed using the pora method with a 15 cm plant-to-plant spacing and a 30 cm row-to-row spacing. The experiment was laid out in a completely randomized block design with three replications and the data have been represented as mean values of the replications. Plants were harvested manually and sun-dried. The seeds were cleaned and separated, and their weight was recorded. The stover yield was calculated by deducting the seed weight from the bundle weight. The seed and stover yields were expressed in kg ha^−1^. 

### 2.4. Estimation of Boron in the Soil Sample

Boron concentration in the soil was estimated by employing the method described by John et al. [27]. In brief, 20 mL of distilled water was added to the flask containing 10 g of the soil sample and left for a few minutes. The sample was heated until it started boiling then this was followed by 5 min of refluxing. The flask was allowed to cool at room temperature and filtered. For the calibration curve, standard solutions of B (10–50 mg L^−1^) in deionized water were prepared using boric acid (H_3_BO_3_). H reagent was added to 1 mL of aliquot (sample and standard solution), 2 mL of buffer and 2 mL of azomethine and allowed to develop in color for 1 h. The concentration of B was estimated spectrophotometrically using Shimadzu (Macro Scientific UV-1800 Model form Australia) UV spectrophotometer, through the formation of a colored complex of B with azomethine H at 420 nm [27].

### 2.5. Estimation of Boron in the Plant Sample

To estimate the B concentration in the plant tissues, the samples were washed successively with tap water, 0.1 N HCl, and deionized water, respectively. The samples were dried at room temperature, which was followed by oven drying at 60 °C and then grinding. The samples were weighed (0.5 g for straw and 1 g for seed) and placed in a muffle furnace for 4 h at 450–500 °C. After cooling the samples at room temperature, deionized water was added to the samples followed by the addition of 10 mL of 6 N HCl. The samples were kept at room temperature for 1 h. Samples were stirred vigorously with a B free glass rod to break the ash particles. The samples were filtered and B concentration was determined spectrophotometrically from the filtrate. To determine B concentration in the plant tissues, the azomethine-H method was followed in the same way that was used to calculate the B concentration in the soil [27]. Boron uptake in the seed and stover was calculated (as given in Equation (1)) by multiplying concentrations (Table A1 in Appendix A) with respective yield as described earlier [28,29].

(1) $$B\;\mathrm{uptake}\;\left({g\;\mathrm{ha}}^{-1}\right)=\frac{B\;\mathrm{concentration}\;\left({\mathrm{mg}\;\mathrm{kg}}^{-1}\right)\times \mathrm{Yield}\left({\mathrm{kg}\;\mathrm{ha}}^{-1}\right)}{1000}$$

### 2.6. Estimation of Certain Quality Parameters

The effect of foliar application of B on antioxidant activity (AOA), theoretical oil content (TOC) and acid value level was estimated. The data of analyzed samples were presented as a mean value of triplicates. Please note that chemicals used in the present study were of AR grade and purchased from Thermo Fisher Scientific.

#### 2.6.1. Estimation of Antioxidant Activity (AOA)

A weighed powdered sample (1.0 g) was taken in a round-bottom volume metric flask and mixed with 20 mL of acidified (0.1% HCl) aqueous methanol (80% *v/v*). The flask was then refluxed at 40 ℃ for about 2 h, which was followed by cooling. The mixed homogenate was transferred to centrifuge tubes and centrifuged at 3000 rpm for 15 min. The supernatant was then transferred to a 50.0 mL volumetric flask. To the residue, 20.0 mL of aqueous methanol was added and the same process was repeated. Finally, the volume of collected extract was made up to 50.0 mL using acidified aqueous methanol. It was then stored in the refrigerator for further analysis [30]. Antioxidant activity was expressed in terms of DPPH (1,1-Diphenyl-2-picrylhydrazyl) radical scavenging activity as described by Sharma et al. [31]. It involved a colorimetric reduction reaction between the methanolic extracts and the DPPH dye solution. To 0.1 mL of methanolic extract, 3.9 mL of freshly prepared DPPH dye solution (0.1 mM) was added, followed by incubation in the dark for 45 min. The absorbance was recorded at 515 nm. In addition, a control was taken for comparison and its absorbance was recorded at zero min. The results were calculated using the following formula as given in Equation (2):

(2) $$\%\;\mathrm{Inhibition}\;\mathrm{of}\;\mathrm{DPPH}\;=1-\frac{{\mathrm{Abs}}_{T}}{{\mathrm{Abs}}_{o}}\times 100$$

where, Abs_T_ is the absorbance of the sample after 30 min; Abs_o_ is the absorbance of the control at 0 min.

#### 2.6.2. Estimation of Total Oil Content (TOC)

Oil content was determined by following the standard protocol of AOAC (2012). For determination of the TOC, 1 g powder seed sample was placed in a thimble of Soxhlet apparatus and the oil was extracted in 150 mL of hexane loaded into a distillation flask. On heating, the solvent evaporated in the flask and condensed successively in the main chamber, thus extracting the oil. After 4 h of extraction, the solvent was removed and the mass of oil was determined by the gravimetric method [32].

#### 2.6.3. Estimation of Acid Value

The estimation of acid value was performed using the acid–base titration method. In brief, the sample was titrated against 0.1 N KOH in hot 2-propanol solution, using phenolphthalein as an indicator, and results were expressed in mg KOH g^−1^ oil. During titration, the acids present in the sample underwent a neutralization reaction with KOH used as the base. The values obtained represent the acid number, which was proportional to the acid value content [33].

### 2.7. Boron Use Efficiency Indices

Boron agronomic efficiency (BAE) and boron crop recovery efficiency (BCRE) were calculated using the following expressions as previously suggested [34].

(3) $$\mathrm{BAE}=\frac{Y_{t}-Y_{c}}{B}$$

Y_t_ and Y_c_ refer to the seed yield (kg ha^−1^) in B-fertilized plots and in the control, and B refers to the B applied (kg ha^−1^).

(4) $$B\;\mathrm{crop}\;\mathrm{recovery}\;\mathrm{efficiency}\;\left(\mathrm{BCRE}\right)=\frac{U_{B}-U_{C}}{B}\times 100$$

U_B_ and U_C_ refer to the total B uptake (kg ha^−1^) in B-fertilized plots and in the control.

### 2.8. Economic Analysis

Prevailing market prices of the inputs were considered for working out the cost of cultivation. However, the sesame seed cost (US$1.60 kg^−1^) and the market rate of sesame for produce (US$1.34 kg^−1^) were used for the calculation of the cost of cultivation. 

$\mathrm{Net}\;\mathrm{Return}\;\left({\mathrm{US}\$\;\mathrm{ha}}^{-1}\right)=\mathrm{Gross}\;\mathrm{return}\;\left({\mathrm{US}\$\;\mathrm{ha}}^{-1}\right)-\mathrm{Cos}t\;\mathrm{of}\;\mathrm{cultivation}\;\left({\mathrm{US}\$\;\mathrm{ha}}^{-1}\right)$ [35]

(5) $$B:C\;\mathrm{ratio}=\frac{\mathrm{Gross}\;\mathrm{return}\;\left({\mathrm{US}\$\;\mathrm{ha}}^{-1}\right)}{\mathrm{Cos}t\;\mathrm{of}\;\mathrm{cultivation}\;\left({\mathrm{US}\$\;\mathrm{ha}}^{-1}\right)}$$

### 2.9. Statistical Analysis

The data were analyzed statistically using the SAS statistical program using one way ANOVA. Duncan’s multiple range test was used for significance comparisons of means at a 0.05 level of probability when the F values were significant in a randomized block design [36]. 

## 3. Results

### 3.1. Effect of B on Seed and Stover Yield of Sesame

In both study years, boron application showed a significant impact on seed and stover yield (Table 3). 

The highest increase of 26.75% in seed yield over the control in the two years was recorded in treatment T8 (706.6 kg ha^−1^) where B was applied at 30 mg L^−1^ at the flowering and capsule formation stages. The result of treatment T8 was statistically at par with treatments T4 (679.5 kg ha^−1^) and T7 (662.4 kg ha^−1^) where B was applied at 30 mg L^−1^ at the flowering stage and at 20 mg L^−1^ at the flowering and capsule formation stages, respectively. Likewise, an increase in stover yield was the highest when using treatment T4 (34.51%) as compared to the control. The stover yield of treatment T4 (4420.7 kg ha^−1^) was statistically at par with treatment T7 (4412.5 kg ha^−1^) and T8 (4293.5 kg ha^−1^). The seed and stover yield was associated with the rate of B application. Both the seed and stover yield initially increased with an increasing rate of B application up to 30 mg L^−1^, it then decreased with an increasing B application rate (T5 and T9). Thus, the results indicated that excessive B application lowered the yield of sesame. Overall, treatments T4, T7 and T8 showed significantly higher results in comparison to the control.

### 3.2. Effect of B on Its Uptake by Seed and Stover of Sesame

The results of the two-year study showed that B uptake in seed and stover of sesame increased significantly with B application at higher levels as compared to the untreated plants (Table 4). 

The mean of the two years’ worth of data demonstrated that the maximum increase in B uptake by seed (64.08%) and stover (70.07%) were recorded in treatment T8 and T4, respectively. The B uptake by seed in treatment T8 (205.40 g ha^−1^) was not statistically different from those of treatment T4 (192.94 g ha^−1^). Thus, B application at 30 mg L^−1^ applied at the flowering and capsule formation stages showed comparable results. The higher dose of B application (40 mg L^−1^) in T5 and T9 significantly reduced the B uptake in sesame (180.28 and 177.25 g ha^−1^, respectively). The water spray in treatment T2 and T6 at the flowering stage and at the flowering and capsule formation stages (131.49 and 133.50, respectively) did not show significant improvement in B uptake compared to the control (125.18 g ha^−1^). Similar results were observed in the stover of sesame for B uptake. The highest B uptake was recorded in T4 (1726.85 g ha^−1^), which was not statistically different from those of treatments T7, T8 and T9.

### 3.3. Effect of B on Certain Quality Parameters of Sesame

The data in Table 5 revealed that the B application improved certain quality parameters of the sesame seed. 

The mean of the two years’ worth of data demonstrated that the AOA of sesame seed recorded significant improvement with B application as compared to the control. The highest AOA was recorded in treatment T8 (69.41%), which was not statistically different from those of treatments T7 (68.65%) and T9 (68.89%). Thus, the rate of B application at similar growth stages did not affect the AOA as the variation observed in treatment T3, T4 and T5 were not significant. Water spray did not affect the AOA as treatments T2 and T6 did not show significant variation as compared to the control.

Boron application improved the TOC of sesame seed to a significant extent as compared to the control. Maximum TOC was recorded in treatment T5 (45.98%), which was significantly at par with treatments T8 (44.35%) and T9 (45.92%). Thus, the rate of B application significantly affected the TOC as significant variation was also observed among the treatments T3, T4 and T5, and likewise in treatments T7, T8 and T9. However, the time of B application did not show a significant effect on the TOC as treatments in which B was applied at the flowering and capsule formation stages showed no significant variation as compared to the treatments in which B was applied at the flowering stage alone at the same rate. Water spray did not affect the TOC as treatments T2 and T6 did not show significant variation as compared to the control. For acid value, the mean of the two years’ worth of data indicated that the effect of B application rate and time of application did not significantly affect the acid value of sesame seed oil.

### 3.4. Effect of B on Efficiency Indices and Economic Analysis

**Figure 1:**
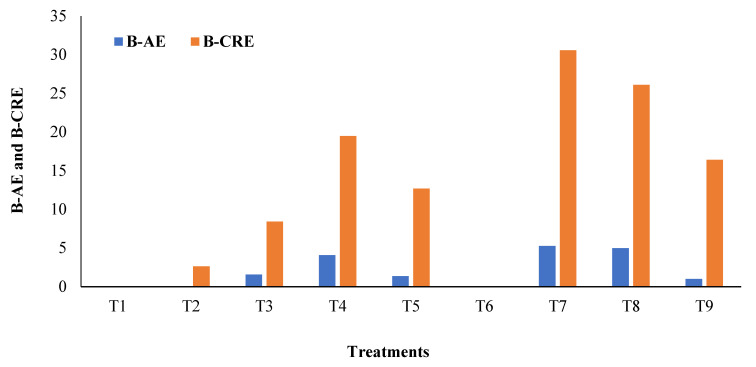
Level and stage of B application on B use efficiency indices of sesame. Experimental treatment details (in the kharif seasons of 2019 and 2020) is available in Table 2. BAE and BCRE indicate boron agronomic efficiency and boron crop recovery efficiency.

Foliar application of B improved the boron agronomic efficiency (BAE) and boron crop recovery efficiency (BCRE) as shown in Figure 1. The highest value of BAE was obtained in treatment T7 (5.25) when B was applied at 20 mg L^−1^ at the flowering and capsule formation stages, which was followed by treatment T8 (4.96) and T4 (4.07) when B was applied at 30 mg L^−1^ at both the growth stages and at the flowering stage alone, respectively. Likewise, the highest BCRE was recorded in treatment T7 (30.56) followed by T8 (26.11) and T4 (19.48). Thus, treatment T7 showed the maximum increase in boron use efficiency indices over the control and water spray.

**Figure 2:**
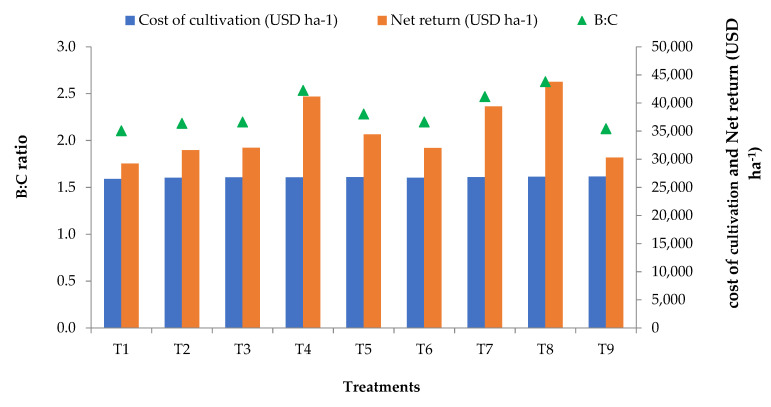
Economic analysis of sesame cultivation affected by different Zn treatments. Experimental treatment details (in the kharif season of 2019 and 2020) is available in Table 2.

The economic analysis demonstrated that the cultivation cost was highest for treatment T9 (US$359.40) whereas net return was highest for treatment T8 (US$584.21) in comparison to the control. Thus, B:C was highest in treatment T8 (2.63). Thus, for maximum economic returns of sesame, treatment T8 can be suggested (Figure 2).

## 4. Discussion

### 4.1. Effect of B on Seed and Stover Yield of Sesame

The data presented in Table 3 demonstrated that the seed and stover yield of sesame increased significantly over the two years in comparison to the control when 30 mg L^−1^ of B was applied at the flowering stage or 20 mg L^−1^ and 30 mg L^−1^ of B was applied at the flowering and capsule formation stages respectively. The B fertilization at 40 mg L^−1^ applied at the flowering and capsule formation stages significantly decreased in mean seed yield. The improvement in seed and stover yield of sesame due to B application was due to its direct role in cell elongation, cell division and biomass accumulation [37]. Studies in the literature have reported that B fertilizer can be attributed to the increased yield and also resulted in the increased seed and stover yield of sesame [38]. Boron application along with the RDF (recommended dose of fertilizers) recorded a higher crop growth rate and relative growth rate, which increased the sesame seed yield over the application of RDF alone [37]. The beneficial effect of B on yield was consistent with the results of others in rice [39], barley [40] and mungbean [41]. The decrease in seed yield at a higher B application rate might be related to the decrease in either cell number or cell width [41]. Similar results of a decrease in yield due to B toxicity at higher concentrations have also been observed in wheat and barley [42,43].

### 4.2. Effect of B on Its Uptake by Seed and Stover of Sesame

A perusal of the data has shown that the mean B uptake by seed and stover of sesame increased significantly compared to the control at 30 mg L^−1^ with one spray applied at the flowering stage and two sprays applied at the flowering and capsule formation stages. The B fertilization at 40 mg L^−1^ applied at the flowering and capsule formation stages significantly decreased the mean seed and stover B uptake. The trend was attributed to the combined effect of yield and B concentration. The increase in B application dose might have increased the B availability and thus increased the uptake. The results are concordant with previous studies where B application increased the B uptake in sesame [35]. At a higher B application rate, although B concentration increased as compared to the lower level, the lower uptake was due to the reduced seed yield. This is evident from studies in the literature where the B application at a lower dose increased the B uptake in sunflower as compared to the control, whereas a higher dose decreased the B uptake [44].

### 4.3. Effect of B on Certain Quality Parameters of Sesame

The data presented in Table 5 revealed that B application significantly enhances the AOA and TOC, whereas the variation observed in the acid value was not significant. The results of AOA were in close agreement with the antioxidant activity of sesame seeds reported by Dravie et al. [45]. The present findings are concordant with the results of studies in the literature where B application enhanced the antioxidant activity of maize cultivars [20]. A similar increase in antioxidant activity with B application has been reported in soybeans [21]. The results also inferred that the B application showed a dose-dependent effect on the TOC of sesame. The results are attributed to the increased B concentration. Similar results have been reported earlier, where oil content in sunflower was significantly increased with increasing B concentration [19].

### 4.4. Effect of B on Efficiency Indices and Economic Analysis

Boron application predominantly affects plant growth at the reproductive stage as compared to the vegetative stage of the crop [46]. An increase in BAE with additional B supply might be attributed to the higher starch utilization, which results in more seed setting and translocation of assimilates to developing grains, thus increasing the crop yield. High BCRE resulting from B application might be due to enhanced B availability, which increased the B uptake and thus BCRE. The cost of cultivation in treatment T9 is highest due to the higher amount of applied B fertilizer (40 mg L^−1^) and twice the number of applications (flowering and capsule formation stages). However, the net return was highest in treatment T8 due to higher yield and a lower amount of applied B fertilizer (30 mg L^−1^) in comparison to treatment T9. Thus, B:C was highest when B fertilizer was applied at 30 mg L^−1^ at the flowering and capsule formation stages due to higher net return and lower cost of cultivation. Similarly, Akshatha and Rajkumar also concluded that the economics of B use in sesame is highly attractive [47]. 

## 5. Conclusions

The results of the present study reflected the beneficial effect of boron application in improving the agroeconomic indicators of sesame crop. During both the years, foliar-applied B at 30 mg L^−1^ at the flowering and capsule formation stages in sandy loam soil showed the maximum increase in seed yield, B uptake and B:C ratio. Moreover, certain oil quality parameters including antioxidant activity and oil content were significantly enhanced with B application at 30 mg L^−1^ at the flowering and capsule formation stages. The efficiency indices viz., BAE and BCRE, were positively affected when B was applied at the rate of 20 and 30 mg L^−1^ at the flowering and capsule formation stages. However, water spray did not significantly affect the agroeconomic indicators. Thus, application of B at 30 mg L^−1^ at the flowering and capsule formation stages can be recommended to enhance grain yield, B uptake, certain quality parameters and high economic returns of sesame cultivation on sandy loam soil. In the future, further studies can be performed to optimize the level of B for sesame cultivation on different types of soils.

## Figures and Tables

**Table 1:** Basic physicochemical analysis of experimental soil before the start of the trial.

Soil Properties	Values	Method
Soil texture	Sandy loam	[22]
pH	7.70	
EC (dSm^−1^)	0.35	
Bulk density (g cm^−3^)	1.42	
Organic matter (%)	0.83	[23]
Total N (%)	0.39	[22]
Available P (kg ha^−1^)	19.66	[24]
Available K (kg ha^−1^)	128.85	[25]
DTPA-extractable Zn (mg kg^−1^)	0.74	[26]
DTPA-extractable Fe (mg kg^−1^)	20.7	
DTPA-extractable Mn (mg kg^−1^)	6.26	
DTPA-extractable Cu (mg kg^−1^)	0.56	
Available B (mg kg^−1^)	0.48	[27]

**Table 2:** Experimental treatment details (kharif season of 2019 and 2020).

Treatments	Source	Boron Application Rate (mg L^−1^)	Crop Stages of B Application	
			Flowering Stage	Capsule Formation Stage
T1	Control	-		−
T2	Water spray	-	+	−
T3	Boron	20	+	−
T4	Boron	30	+	−
T5	Boron	40	+	−
T6	Water spray	-	+	+
T7	Boron	20	+	+
T8	Boron	30	+	+
T9	Boron	40	+	+

**Table 3:** Level and stage of B application on seed and stover yield of sesame.

Treatments	Seed Yield (kg ha^−1^)				Stover Yield (kg ha^−1^)			
	1st Year	2nd Year	Mean	% Increase	1st Year	2nd Year	Mean	% Increase
T1	548.1 ^b^ ± 42.7	566.3 ^b^± 7.0	557.2 ^b^	-	3065.4 ^b^ ± 168.7	3506.8 ^c^ ± 260.1	3286.6 ^c^	-
T2	587.6 ^b^ ± 2.0	579.2 ^b^ ± 15.2	583.4 ^b^	4.67	3083.2 ^b^ ± 180.1	3761.6 ^bc^ ± 246.1	3422.4 ^bc^	4.14
T3	533.3 ^b^ ± 39.2	643.1 ^ab^ ± 25.5	588.2 ^b^	5.57	3238.7 ^b^ ± 204.5	3976.3 ^bc^ ± 238.1	3607.5 ^bc^	9.77
T4	700.2 ^ab^ ± 25.1	658.8 ^a^ ± 24.6	679.5 ^a^	21.90	4065.9 ^ab^ ± 288.8	4775.5 ^a^ ± 289.5	4420.7 ^a^	34.51
T5	612.4 ^b^ ± 13.69	611.6 ^ab^ ± 16.0	612.5 ^b^	9.87	3230.1 ^b^ ± 163.6	4013.7 ^b^ ± 201.5	3621.4 ^bc^	10.19
T6	576.5 ^b^ ± 11.3	598.3 ^b^ ± 17.3	587.4 ^b^	5.39	3113.5 ^b^ ± 140.7	4007.9 ^b^ ± 214.0	3560.7 ^bc^	8.34
T7	663.1 ^ab^ ± 13.0	661.7 ^a^ ± 35.6	662.4 ^a^	18.85	4068.8 ^ab^ ± 552.4	4756.2 ^a^ ± 169.9	4412.5 ^a^	34.27
T8	755.7 ^a^ ± 207	656.5 ^a^ ± 10.7	706.6 ^a^	26.75	4375.6 ^a^ ± 103.3	4212.4 ^b^ ± 109.1	4293.5 ^ab^	30.65
T9	556.2 ^b^ ± 60.6	588.4 ^b^ ± 50.9	572.3 ^b^	2.69	3552.3 ^b^ ± 105.8	4048.1 ^b^ ± 245.1	3800.2 ^b^	15.64
LSD ≤ 0.05	133.4	51.2	80.1	-	737.5	452.4	501.3	-

The treatments detailed are in Table 2. Values with the same superscript letter do not differ significantly at the 5% level using Duncan’s multiple range test.

**Table 4:** Level and stage of B application on B uptake by seed and stover of sesame.

Treatments	Seed B Uptake (g ha^−1^)				Stover B Uptake (g ha^−1^)			
	1st Year	2nd Year	Mean	% Increase	1st Year	2nd Year	Mean	% Increase
T1	123.67 ^c^ ± 11.47	126.67 ^c^ ± 2.94	125.18 ^c^	-	955.29 ^c^ ± 73.89	1074.26 ^d^ ± 66.32	1015.36 ^d^	-
T2	132.85 ^c^ ± 5.38	130.12 ^c^ ± 1.95	131.49 ^c^	5.04	963.04 ^c^ ± 78.76	1158.65 ^d^ ± 61.64	1061.57 ^cd^	4.55
T3	136.84 ^c^ ± 12.28	157.89 ^b^ ± 4.21	147.67 ^c^	17.97	1146.33 ^bc^ ± 109.78	1337.52 ^c^ ± 52.98	1245.19 ^c^	22.64
T4	200.91 ^ab^ ± 7.61	185.09 ^a^ ± 6.67	192.94 ^ab^	54.13	1609.32 ^ab^ ± 118.80	1840.72 ^a^ ±116.11	1726.85 ^a^	70.07
T5	185.94 ^b^ ± 11.58	174.63 ^a^ ± 8.57	180.28 ^b^	44.02	1353.40 ^b^ ± 76.39	1571.92 ^b^ ± 46.94	1468.01 ^b^	44.58
T6	131.92 ^c^ ± 7.76	135.04 ^c^ ± 4.78	133.50 ^c^	6.65	984.23 ^c^ ± 42.66	1239.58 ^c^ ± 58.18	1113.43 ^cd^	9.66
T7	179.40 ^b^ ± 17.75	165.29 ^b^ ± 14.31	172.33 ^b^	37.67	1518.98 ^ab^ ± 101.89	1629.09 ^b^ ± 6.30	1579.35 ^ab^	55.55
T8	227.15 ^a^ ± 21.14	184.62 ^a^ ± 3.95	205.40 ^a^	64.08	1816.22 ^a^ ± 68.46	1623.10 ^b^ ± 143.79	1718.45 ^a^	69.25
T9	174.22 ^bc^ ± 18.33	180.17 ^ab^ ± 17.42	177.25 ^b^	41.60	1537.11 ^ab^ ± 72.27	1699.15 ^b^ ± 113.55	1619.75 ^ab^	59.52
LSD ≤ 0.05	39.54	16.25	23.75		301.25	146.74	199.10	

The treatments detailed are in Table 2. The values with the same superscript letter do not differ significantly at the 5% level when using Duncan’s multiple range test.

**Table 5:** Level and stage of B application on AOA, TOC and acid value in sesame grain.

Treatments	AOA (%) *	TOC (%) **	Acid Value
T1	65.21 ^c^ ± 0.27	34.20 ^d^ ± 1.06	0.87 ± 0.06
T2	65.59 ^c^ ± 0.32	34.72 ^d^ ± 0.71	0.87 ± 0.06
T3	68.25 ^b^ ± 0.87	37.35 ^c^ ± 0.98	0.86 ± 0.05
T4	68.45 ^b^ ± 0.69	42.15 ^b^ ± 0.18	0.88 ± 0.04
T5	68.40 ^b^ ± 0.24	45.98 ^a^ ± 0.92	0.88 ± 0.03
T6	65.63 ^c^ ± 0.20	34.83 ^d^ ± 0.67	0.89 ± 0.05
T7	68.65 ^ab^ ± 0.74	39.57 ^c^ ± 3.77	0.90 ± 0.02
T8	69.41 ^a^ ± 0.42	44.35 ^ab^ ± 0.77	0.89 ± 0.01
T9	68.89 ^ab^ ± 0.36	45.92 ^a^ ± 0.38	0.89 ± 0.01
LSD ≤ 0.05	0.87	2.38	NS

* AOA, antioxidant activities; ** TOC, total oil content. The values with the same superscript letter do not differ significantly at the 5% level when using Duncan’s multiple range test.

## Data Availability

Data recorded in the current study are available in all Tables and Figures of the manuscript.

## Appendix A

**Table A1:** Effect of level and stage of B application on B concentration of seed and stover of sesame.

Treatments	Seed B Concentration (mg kg^−1^)			Stover B Concentration (mg kg^−1^)		
	1st Year	2nd Year	Mean	1st Year	2nd Year	Mean
T1	22.59 ^d^ ± 1.22	22.37 ^d^ ± 0.69	22.48 ^f^	31.17 ^d^ ± 1.69	30.64 ^d^ ± 0.94	30.90 ^e^
T2	22.63 ^d^ ± 0.98	22.49 ^d^ ± 0.29	22.56 ^f^	31.23 ^d^ ± 1.35	30.80 ^d^ ± 0.40	31.02 ^e^
T3	25.66 ^c^ ± 1.08	24.55 ^c^ ± 0.86	25.11 ^e^	35.41 ^c^ ± 1.48	33.64 ^c^ ± 1.18	34.52 ^d^
T4	28.69 ^b^ ± 1.27	28.14 ^b^ ± 0.07	28.41 ^c^	39.59 ^b^ ± 1.75	38.55 ^b^ ± 0.10	39.07 ^b^
T5	30.36 ^a^ ± 0.23	28.60 ^b^ ± 0.66	29.48 ^b^	41.90 ^a^ ± 0.32	39.17 ^b^ ± 0.91	40.54 ^b^
T6	22.91 ^d^ ± 0.80	22.58 ^d^ ± 0.21	22.75 ^f^	31.61 ^d^ ± 1.11	30.93 ^d^ ± 0.29	31.27 ^e^
T7	27.06 ^c^ ± 0.46	25.01 ^c^ ± 0.83	26.03 ^d^	37.34 ^c^ ± 0.64	34.26 ^c^ ± 1.13	35.80 ^c^
T8	30.09 ^ab^ ± 0.01	28.13 ^b^ ± 0.27	29.11 ^bc^	41.52 ^ab^ ± 0.02	38.53 ^b^ ± 0.37	40.03 ^b^
T9	31.36 ^a^ ± 0.66	30.64 ^a^ ± 0.29	31.00 ^a^	43.28 ^a^ ± 0.91	41.97 ^a^ ± 0.39	42.63 ^a^
LSD ≤ 0.05	1.50	0.96	0.88	2.07	1.32	1.21

The values with the same superscript letter do not differ significantly at the 5% level when using Duncan’s multiple range test.
